# Awareness data on cervical cancer among females of rural and urban areas of Haryana, India

**DOI:** 10.1016/j.dib.2024.110168

**Published:** 2024-02-07

**Authors:** Ritu Yadav, Meenakshi B. Chauhan, Chetna Yadav, Shalu Ranga, Parul Ahuja, Mukesh Tanwar, Nikita Balhara, Lokesh Kadian, Preeti Chauhan, Neha Tanwar, Chavi Ahlawat

**Affiliations:** a Department of Genetics, Maharshi Dayanand University, Rohtak, Haryana 124001 India; b Department of Obstetrics and Gynecology, Pandit Bhagwat Dayal Sharma University of Health Sciences, Rohtak, Haryana 124001 India; c School of Medicine, Indiana University, Indianapolis, IN 46202 United States; d Department of Biotechnology, Chandigarh Group of Colleges, Landran, Mohali, Chandigarh 140307 India

**Keywords:** Rural and urban population, Awareness, Knowledge, Cervical cancer, Risk factors, HPV

## Abstract

A cross-sectional study was done to assess the degree of current awareness and behaviors about cervical cancer among females in urban and rural areas of North India. This survey was conducted on one thousand females (500 rural and 500 urban). A well-structured questionnaire was designed to collect information about participants’ knowledge on cancer of cervix uteri such as age, height and weight measurements, marital status, menstrual status, personal hygiene, age at menarche, sexual history, pregnancy and abortion history, use of contraceptive pills for birth-control, smoking, alcohol consumption, and other relevant information. The data was collected by conducting face-to-face interviews after obtaining the verbal consent of the participants. The data has the potential to reduce disease burden by spreading awareness about symptoms and risk factors of cervical cancer as well as implementation of effective early screening strategies.

Specifications Table

**Table:** 

Subject	Cancer Research
Specific subject area	Cervical cancer awareness assessment
Data format	Raw, filtered
Type of data	Table, numbers, XLSX, percentages, mean, median and standard deviations
Data collection	A questionnaire was designed to collect data on cervical cancer such as age, height and weight measurements, marital status, menstrual status, personal hygiene, age at menarche, sexual history, pregnancy and abortion history, use of contraceptive pills for birth-control, smoking, alcohol consumption, and other relevant information. The responses of women who were questioned in the previous study by Kadian et al. were excluded [2]. Both married as well as unmarried women aged between 18-70 years were included in the study.
Data source location	Site of data collection: Rural and urban areas of Haryana Site of data storage: M.D. University Rohtak (Haryana)- India
Data accessibility	Repository name: Mendeley Data Data Identification Number: 10.17632/xxdfkvwyx9.1 URL: https://data.mendeley.com/datasets/xxdfkvwyx9/1
Related research article	Kadian L, Gulshan G, Sharma S, Kumari I, Yadav C, Nanda S, Yadav R. A Study on Knowledge and Awareness of Cervical Cancer Among Females of Rural and Urban Areas of Haryana, North India. J Cancer Educ. 2021 Aug;36(4):844-849. doi: 10.1007/s13187-020-01712-6. PMID: 3211236.

## 1 Value of the Data

• Awareness data collection on cervical cancer contributes to the profound need for awareness programs in both rural and urban areas. Every individual needs to know that mortality due to cervical cancer can be extensively prevented if get screened at earlier stages.
• Disease burden can be decreased by offering health education and the implementation of effective early screening. In developing countries like India, low cost of screening and treatment is a requisite. This serves as a call for action of authorities, which should provide need-based policy design including a recognised health care system, cost-effective screening and treatment.
• The current data will pave the way for further independent investigations, comparison to other studies, and database consolidation with supplementary resources so that the incidence of cervical cancer can be reduced in developing nations. Furthermore, the findings may be extended to meta-analyses and replication studies as well.

## 2 Background

Worldwide, cervical cancer represents a significant health burden for women. While developed countries have successfully reduced their incidence through the enactment of cytology-based screening programs, cervical cancer remains a significant cause of death from cancer among females in developing nations including India [4]. Globally, cervical cancer (6.5%) is the fourth most common cancer type in females after breast cancer (24.5%), colorectum cancer (9.4%) and lung cancer (8.4%) (GLOBOCAN, 2020). The year 2020 accounted for 604,100 new cases and 341,831 deaths due to cervical cancer all over the world [5].

India accounts for about one-fourth of all cervical cancer deaths worldwide, with a disproportionately high prevalence among females residing in rural regions and those having low-income status [6]. The reason for the high mortality rate in India is the low level of awareness regarding cervical cancer symptoms, screening programs, risk factors and preventive measures [2].

To reduce the mortality rates, timely diagnosis and effective treatment both are crucial. In India, most of the cervical cancer cases are usually diagnosed at later stages, resulting in lower survival rates. Routine screening procedures including Pap (Papanicolaou) smear, HPV-DNA testing and cervical visual inspection with acetic acid (VIA) have effectively lowered the occurrence and death rates of cervical cancer in developed nations[1]. Unfortunately, developing countries face challenges in the implementation of effective screening and prevention programs due to financial, social, and logistical barriers. India also lacks nationwide screening programs for cervical cancer due to limited awareness, misconceptions about gynaecological diseases and the absence of comprehensive national policies [3].

The present study aimed at to check the level of awareness among rural and urban female participants of Haryana. Possible relevant factors which were not included and asked by the participants out of their curiosity during the conduction of the previous study [2] are also included in the present study questionnaire. It may help to reduce their misconceptions about gynaecological diseases and early screening, thereby reducing mortality ultimately.

## 3 Data Description

**Fig. 1:**
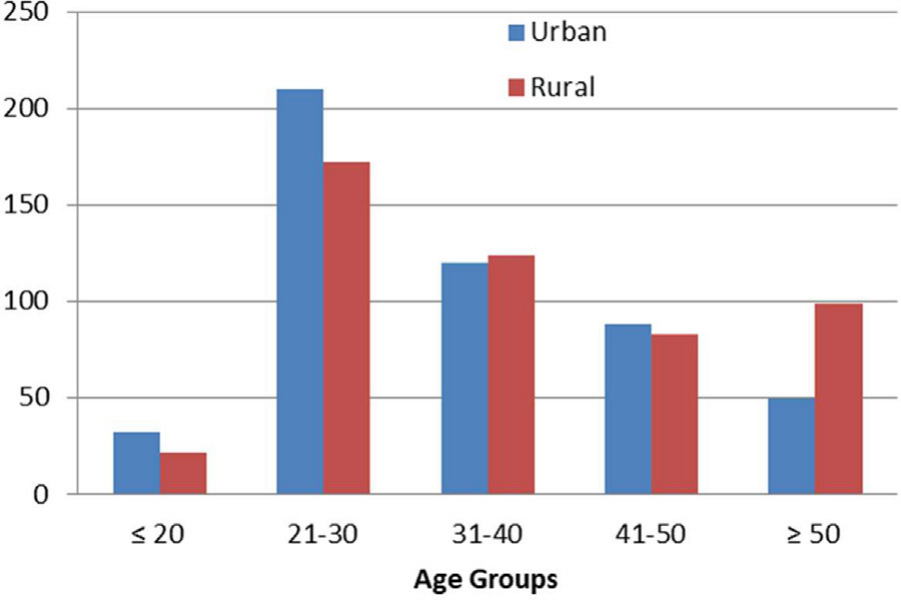
Age groups of participants in the rural and urban areas.

In this survey, data from one thousand females (500 urban and 500 rural) was collected to study the extent of awareness about the risk factors, symptoms and other characteristics of cervical cancer. Table 1 shows the sociodemographic characteristics of the study population. The female participants involved in the survey belonged to various age groups (ranging from 18 to 70 years). Majority of the participants of urban and rural backgrounds were between the age group 21–30 years (42% and 34% respectively) (Fig. 1). The mean age of the urban participants was 34.05 ± 11.96 (median was 31 with the age ranging from 18 to 70 years) and that of urban participants was 37.76 ± 14.48 (median was 35 with the age ranging from 18 to 92 years).

**Table 1:** Sociodemographic characters of participating women from urban and rural areas.

Socio-demographic characteristics	Urban	Percentage (%)	Rural	Percentage (%)
Marital status				
Married	420	84	396	79
Divorced	7	1	1	0
Single	52	10	50	10
Widowed	21	4	53	11
Occupation				
Non-working	320	64	399	80
Student	43	8.6	50	10
Working	137	27.4	51	10

**Fig. 2:**
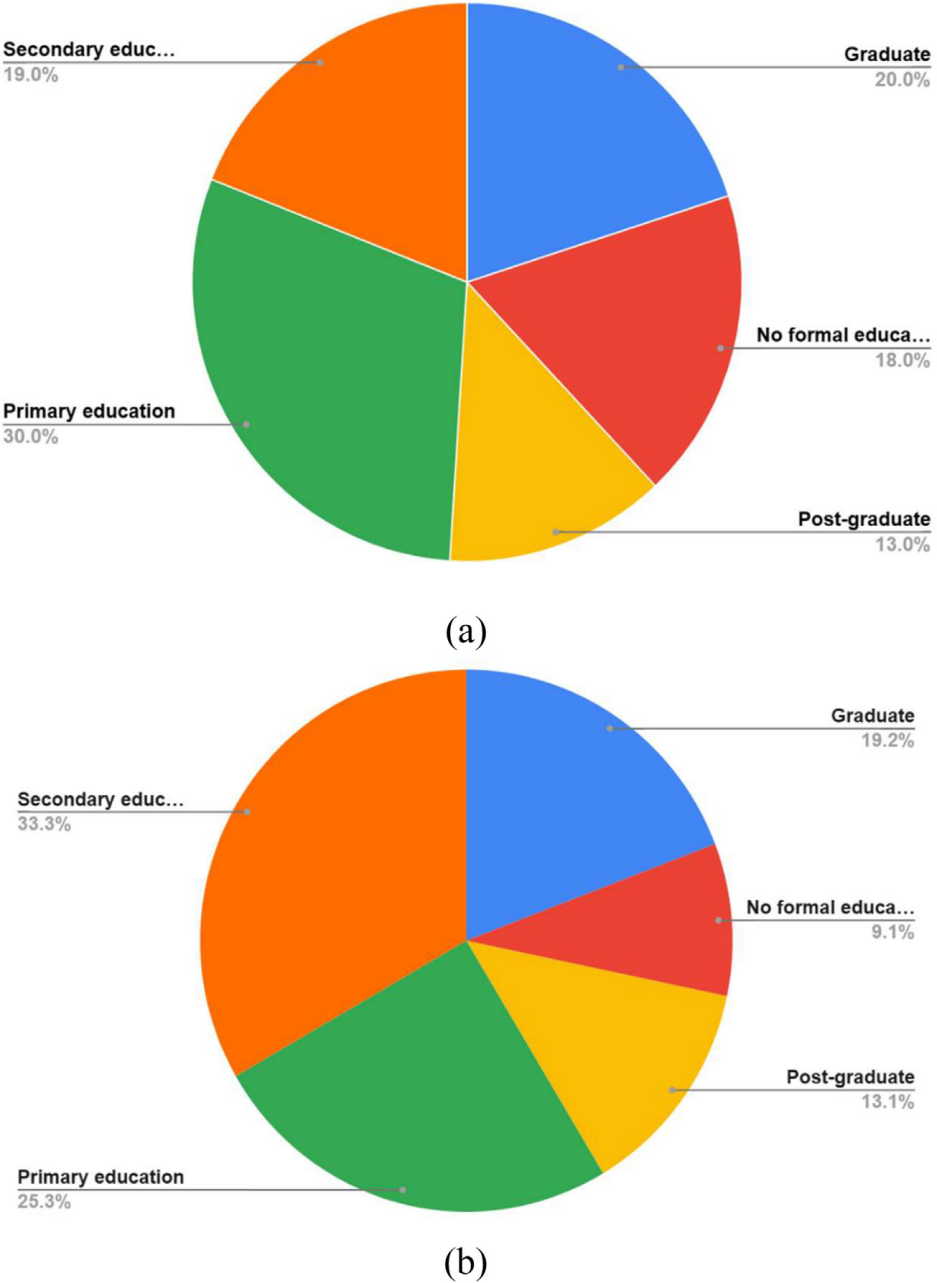
Educational status of enrolled women in (a) rural areas (b) urban areas.

Most of the urban participants had secondary education and rural participants had primary education (33% and 30% respectively) (Fig. 2). Maximum enrolled participants in both urban (84%) and rural (79%) areas were married and a majority of them were non-working from occupation (80% and 64% respectively) (Table 1).

In the present study, females were categorized on the basis of body mass index (BMI) according to CDC (Centers for Disease Control and Prevention) criteria (https://www.cdc.gov/healthyweight/assessing/bmi/adult_bmi/index.html). More than half of urban women were average weight and about 24% were overweight. 22% of urban women were sleep-deprived and under stress or depression. The majority of urban women were not performing any kind of physical activity and good at maintaining personal hygiene. 86% of urban women used sanitary pads as menstrual products. 87% used to wash their vagina regularly after each intercourse. In this data, the most commonly known cancers were breast cancer followed by blood cancer. In rural women, blood cancer was the most commonly known cancer and in urban women breast cancer was the most commonly known cancer.

The maximum females of urban and rural backgrounds had the knowledge that cancer can affect any organ of the body (86% and 92% respectively). Most of the participants in urban (71%) and rural areas (63%) of the present study were not aware of the cancer of cervix. Majority of the females (88% urban and 85% rural) did not know cervical cancer risk factors (Table 2). Family/Friends/Relatives were the main information sources of cervical cancer.

**Table 2:** Basic knowledge of cervical cancer and its screening among urban and rural regions.

Variables	Urban	Percentage (%)	Rural	Percentage (%)	*p* value
Do you know about cancer?					
Yes	400	80	217	43.4	< 0.0001
No	100	20	283	56.6	
Do you know that cancer can affect any organ of the body?					
Yes	429	85.8	458	91.6	0.0041
No	71	14.2	42	8.4	
Have you ever heard about cancer of cervix/cervical cancer?					
Yes	143	28.6	185	37	0.004
No	357	71.4	315	63	
If yes, then do you know that only women are affected?					
Yes	312	62.4	279	55.8	0.0336
No	188	37.6	221	44.2	
Do you know about the signs and symptoms of this cancer?					
Yes	230	46	265	53	0.040
No	270	54	235	47	
Do you have any knowledge about the risk factors of cervical cancer?					
Yes	56	11.2	71	14.2	0.1551
No	444	88.8	429	85.8	
What was the source of information?					
Family/Friends/Relatives	372	74	286	57	<0.0001
Internet	22	4	45	9	
Television	36	7	79	16	
Education	70	14	90	18	

Significance level, p<0.05 (Performed odd ratio at 95% Confidential interval and chi square test)

Most of the females did not have any cancer history. 28% of urban women showed a history of cancer in the family (mother), especially breast cancer history. 12% of rural women had a blood cancer history in their relatives (Grandfather). Only 1.2% of urban women had displayed some symptoms of breast cancer and 1% of rural women had displayed some symptoms of uterine cancer in their own history. From the personal information of the participants, most of the urban and rural women were found to belong to the 13–17 years of age group when they had their first menstruation (89% and 88% respectively).

Mostly all the participants from rural and urban areas had regular menstruation (once in a month) with no pain and normal flow. Rural women with painful menstrual flow reported 1–2 days of pain (66%) while urban women didn't respond to this information. More than half of the total participants in urban (71%) and rural (78%) areas had undergone full-term pregnancy with an average of two children (33% and 37% respectively). Most of the rural women were in the 19–30 years of age group and most of the urban women were above 30 years of age at the time of their first childbirth. There was a gap of 1–2 years between first and second child in the urban participants and 2–3 years in the women of rural areas. A major fraction of the urban and rural women had no history of using any kind of oral contraceptive pills (73% and 81%) and had never undergone hormonal therapy (98% and 92%) (Table 3).

**Table 3:** Personal information of participating urban and rural women.

Variables	Urban	Percentage (%)	Rural	Percentage (%)
Age at menarche				
≤12	41	8.2	45	9
≥18	13	2.6	16	3.2
13–17	444	88.8	439	87.8
Absence of menstrual cycle	2	0.4	0	0
Menstrual cycle status				
Irregular	95	19	60	12
Regular	405	81	440	88
Painful menstruation				
Yes	230	46	180	36
No	270	54	320	64
If painful, then pain lasts up to how many days?				
All days of periods	87	17.4	121	24.2
Day 1–2	146	29.2	330	66
Not responded	266	53.2	49	9.8
Menstrual flow				
Excessive	86	17.2	55	11
Normal	414	82.8	441	88.2
less	0	0	4	0.8
If excessive, then total days for which bleeding lasts?				
1 week	253	50.6	320	64
More than a week	247	49.4	180	36
Bleeding time in a month				
Once in a month	479	95.8	285	57
Twice in a month	21	4.2	215	43
Full term pregnancy				
Age at first child birth				
≤18	106	21.2	43	8.6
19–30	241	48.2	296	59.2
above 30	219	43.8	161	32.2
Use of oral contraceptive pills				
No	364	72.8	402	80.4
Rarely	80	16	33	6.6
Yes, for a longer period	56	11.2	18	3.6
Hormonal therapy				
Hormonal replacement therapy	9	1.8	40	8
No hormonal therapy	491	98.2	460	92

In sexual history, more than half of urban and rural women had never reported bleeding or felt pain during intercourse (78% and 88% respectively). Most of the participants from urban and rural areas became sexually active after the age of 18 years (64% and 66%) and a majority of them were found to use no protection during intercourse (72% and 68%). Maximum number of urban and rural participants had experienced some kind of vaginal infection (92% and 97%) in their lifetime. Husbands of 85% of rural women had certain kinds of infections in their reproductive organs, whereas most of the urban females didn't respond to this information. Most of the participants from urban and rural areas didn't use to smoke or consume alcohol or tobacco. Husbands of 61% of urban women used to consume alcohol on a daily basis, whereas husbands of rural women didn't have a habit of consuming alcohol (Table 4).

**Table 4:** Sexual history of participating urban and rural women.

Variables	Urban	Percentage (%)	Rural	Percentage (%)
Pain or bleeding during intercourse				
No pain and bleeding	387	77.4	437	87.4
Only bleeding	13	2.6	2	0.4
Only pain	47	9.4	9	1.8
Pain and bleeding both	22	4.4	5	1
Didn't respond	31	6.2	47	9.4
Onset of sexual activity				
<18	150	30	121	24.2
≥18	320	64	330	66
Sexually inactive	30	6	49	9.8
Intercourse without protection				
Yes	359	71.8	340	68
No	104	20.8	84	16.8
Sexually inactive	31	6.2	54	10.8
Didn't respond	6	1.2	22	4.4
Vaginal infection				
Yes	36	7.2	14	2.8
No	464	92.8	486	97.2
Does your partner/husband have any kind of infection?				
Yes	1	0.2	4	0.8
No	38	7.6	426	85.2
Didn't respond	461	92.2	70	14
Smoking				
Yes	20	4	6	1.2
No	480	96	494	98.8
Alcohol consumption				
Yes	0	0	1	0.2
No	500	100	499	99.8
Tobacco usage				
Yes	10	2	5	1
No	490	98	495	99
Does your partner/husband smoke or consume alcohol on a regular basis?				
Yes	303	60.6	57	11.4
No	99	19.8	401	80.2
Didn't respond	98	19.6	42	8.4

**Fig. 3:**
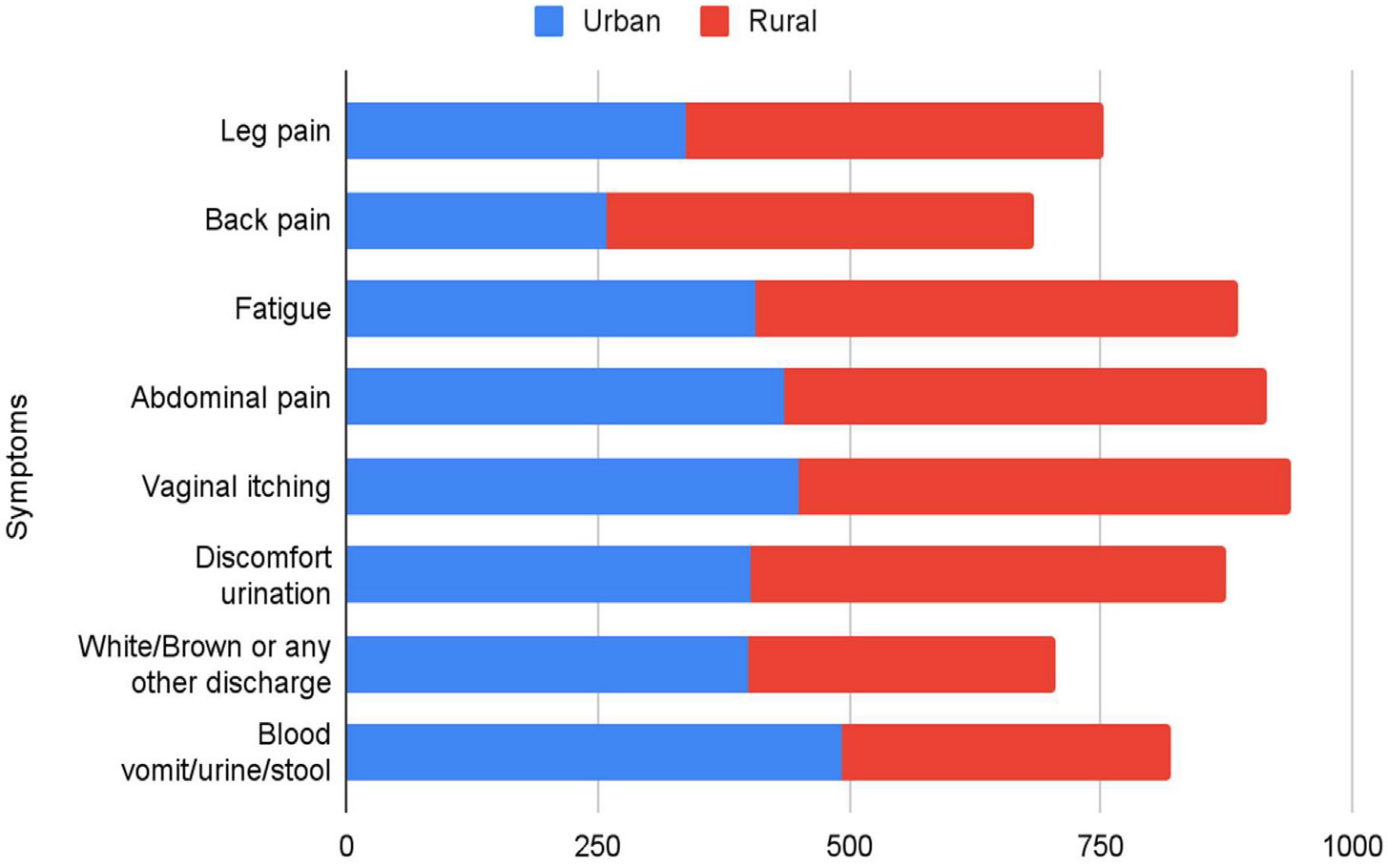
Knowledge of cervical cancer symptoms in participating urban and rural women.

The most commonly known symptoms in rural females were leg pain (83%), back pain (85%), fatigue (96%), vaginal itching (98%), discomfort during urination (95%) and abdominal pain (87%), white/brown or any other discharge (80%) and blood in vomit/urine/stool (98.4%) in the urban females. Lack of knowledge of cervical cancer is well reflected in the low level of understanding of symptoms as the participants who lacked basic education could recognise only two to three symptoms (Fig. 3).

As indicated in the present survey, information about the presence of health facilities and medical care was good in both urban and rural areas as perceived by the participants themselves. Half of the total rural participants never visited the doctor for any counseling about cervical cancer. However, urban participants have visited the medical facilities once in 6 months for counseling purposes. Majority of the urban participants (94%) were not even aware of the HPV vaccine and the appropriate age of getting the vaccine, whereas 73% of rural participants didn't respond to this question.

Half of the urban participants had knowledge of pap smear tests. More than half of urban women were not aware of the fact that having a first-degree relative already affected by this cancer also increases the chances of developing this malignancy. After completion of the survey, most of the urban participants found that they were at the risk of cervical cancer to some extent. 54% of urban participants could conclude that cervical cancer is preventable and curable if diagnosed at earlier stages, however, rural women didn't respond to this information (Table 5).

**Table 5:** Information about medical care.

Variables	Urban	Percentage (%)	Rural	Percentage (%)
Health facilities in your area				
Good	428	85.6	335	67
Poor	3	0.6	60	12
Satisfactory	69	13.8	105	21
Medical care				
Appropriate	237	47.4	395	79
Little	193	38.6	103	20.6
No care	70	14	2	0.4
Delay in medical care				
Yes	78	15.6	12	2.4
No	422	84.4	488	97.6
Visit to a doctor				
Do not visit	139	27.8	254	50.8
Once in 6 months	167	33.4	191	38.2
Once in a month	146	29.2	42	8.4
Twice in a month	48	9.6	13	2.6
Have you heard about the HPV vaccine?				
Yes	27	5.4	23	4.6
No	473	94.6	115	23
Not answered	0	0	362	72.4
If yes, then do you know about the appropriate age for getting the HPV vaccine?				
Yes	11	2.2	14	2.8
No	489	97.8	122	24.4
Not answered	0	0	364	72.8
Have you taken this vaccine?				
Yes	42	8.4	2	0.4
No	458	91.6	135	27
Not answered	0	0	363	72.6
Have you heard about a pap smear test?				
Yes	258	51.6	124	24.8
No	155	31	14	2.8
May be	87	17.4	362	72.4
Do you know that having a first-degree relative already affected by this cancer also increases your risk of developing this cancer?				
May be	180	36	45	9
Yes	15	3	20	4
No	305	61	72	14.4
Not answered	0	0	363	72.6
After answering the survey questions, do you think that you are at risk to some extent?				
Maybe	0	0	2	0.4
Yes	274	54.8	1	0.2
No	226	45.2	131	26.2
Not answered	0	0	366	73.2
Do you think that cervical cancer is preventable/curable if diagnosed at early stages?				
Yes	270	54	81	16.2
No	226	45.2	52	10.4
Not answered	4	0.8	367	73.4

Our key findings include that despite just 30% of all rural women having completed primary education, they are more knowledgeable about cervical cancer than urban women in the following two aspects:

• A comparatively higher number of rural women (37%) have heard about cervical cancer as compared to 28.6% of urban women.
• More women in rural areas were aware about the warning symptoms and signs associated with cervical cancer (53%) as compared to 46% of urban women.

## 4 Experimental Design, Materials and Methods

**Fig. 4:**
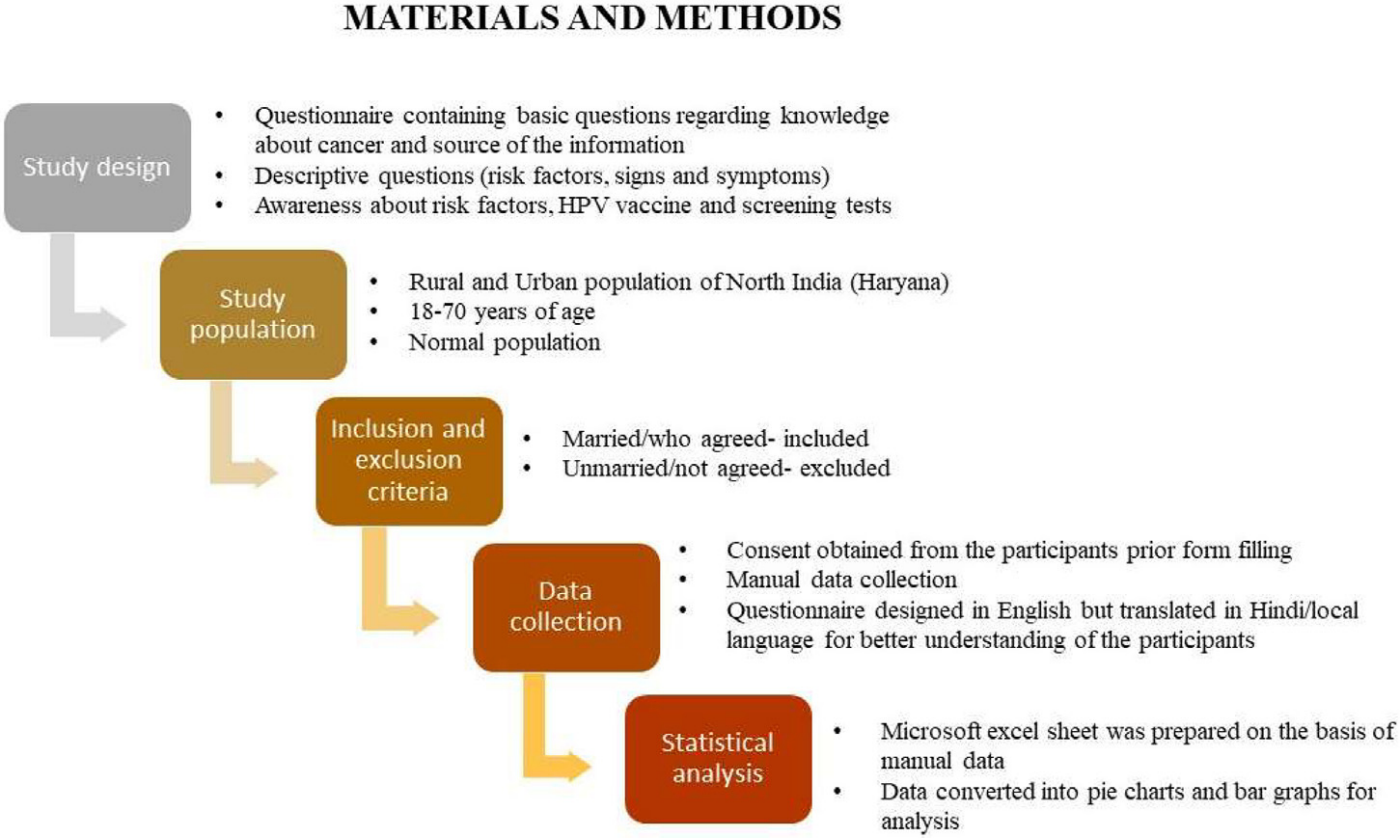
Methodology followed to conduct the present study.

An outline of the methodology used is given in Fig. 4.

### 4.1 Study design and population

One thousand females (500 urban and 500 rural) were selected to assess the level of awareness and knowledge of cervical cancer. The study was conducted in various urban and rural areas of Haryana excluding the areas covered in our previous study [2]. In continuation with the previously conducted study, this survey was carried out in two sessions with a modified questionnaire additionally having dietary and hormonal factors which were not included in the previous study questionnaire. In the first session, no scientific information about factors was provided to the participants to check their level of awareness. In the second session, after checking the awareness level, researchers provided the necessary information to them about the signs and symptoms of cervical cancer and also the need of regular visits to doctors for counseling purposes as well as early screening of the disease.

### 4.2 Exclusion and inclusion criteria

The responses of women who were questioned in the previous study by Kadian et al. were excluded [2]. Both married as well as unmarried women aged between 18 and 70 years were included in the study. All the females voluntarily participated in the survey and were willing to be questioned.

### 4.3 Questionnaire

A well-structured questionnaire was framed to collect information from participants on cervical cancer such as age, marital status, educational status, occupation, family history, gynecological factors (menstrual history, pregnancy history, age at menarche and menopause, abortion history, use of birth-controlling contraceptive pills, HRT), sexual intercourse related factors, lifestyle-related factors (consumption of alcohol, smoking, tobacco usage, height and weight measurements, BMI), personal hygiene, symptoms of cervical cancer and other relevant factors.

The participants were requested to answer the questions included in the questionnaire to assess their knowledge level. The information was collected by conducting face-to-face interviews after obtaining the verbal consent of the participants. The obtained information is the perception of participants which was assessed to find the need of awareness about cervical cancer.

### 4.4 Statistical analysis

This data was analyzed statistically by using Microsoft Excel and online Medcalc software (v17.2). Awareness level about cervical cancer was assessed by applying chi-square test and odds ratio with a 95% confidence level. *p* < 0.05 was set as the level of statistical significance.

## CRediT authorship contribution statement

**Ritu Yadav:** Supervision, Conceptualization, Validation, Methodology, Writing – review & editing. **Meenakshi B. Chauhan:** Conceptualization, Methodology, Writing – review & editing, Visualization. **Chetna Yadav:** Conceptualization, Methodology, Visualization. **Shalu Ranga:** Conceptualization, Methodology, Visualization. **Parul Ahuja:** Conceptualization, Methodology, Visualization. **Mukesh Tanwar:** Conceptualization, Methodology, Writing – review & editing, Visualization. **Nikita Balhara:** Conceptualization, Methodology, Writing – original draft. **Lokesh Kadian:** Conceptualization, Methodology, Visualization. **Preeti Chauhan:** Conceptualization, Methodology, Writing – review & editing, Visualization. **Neha Tanwar:** Conceptualization, Methodology, Writing – original draft. **Chavi Ahlawat:** Conceptualization, Methodology, Writing – original draft.

## Data Availability

Awareness about cervical cancer in rural and urban populations of haryana (Original data) (Mendeley Data).
